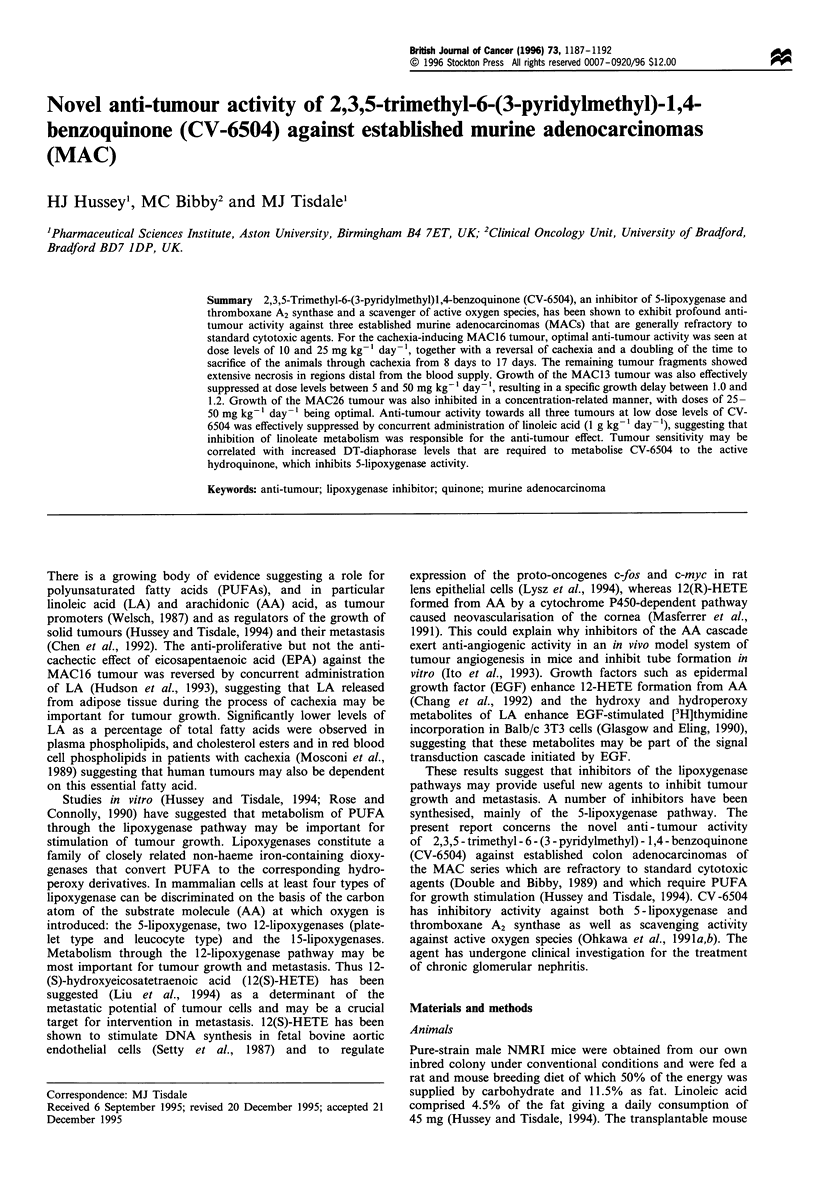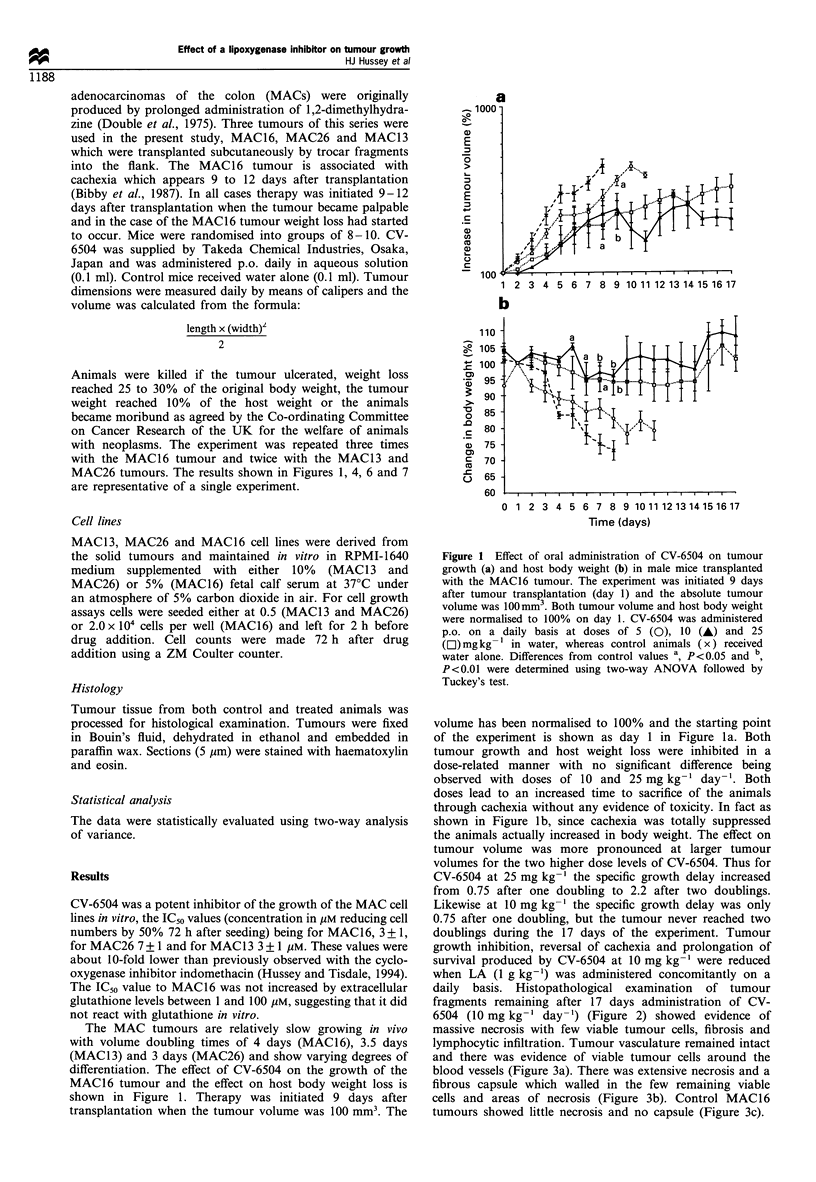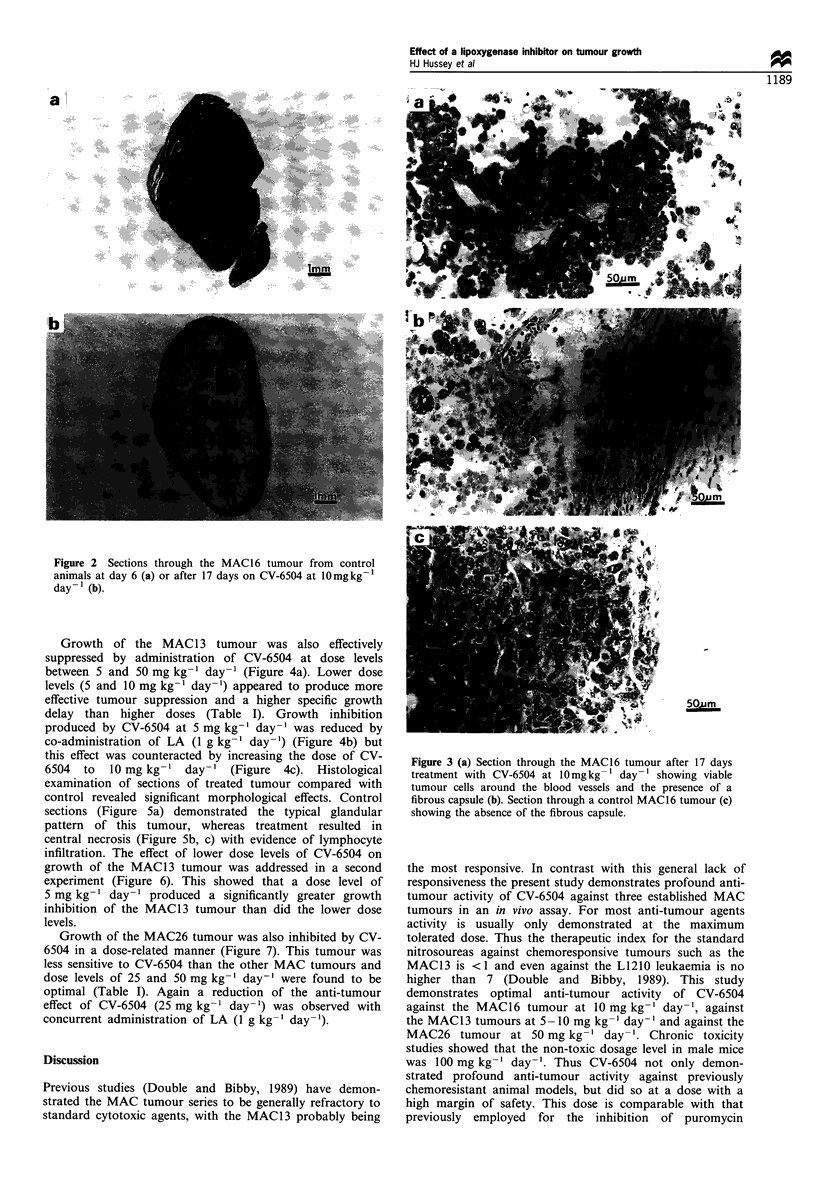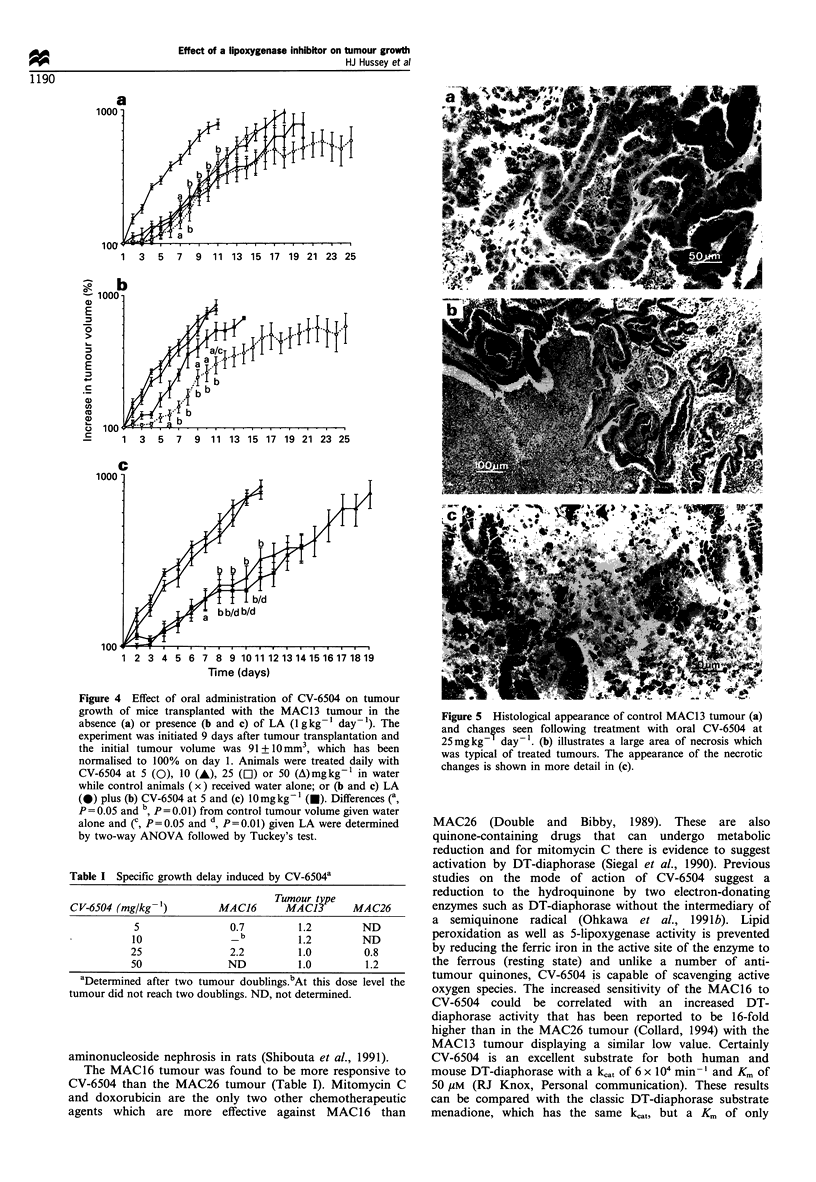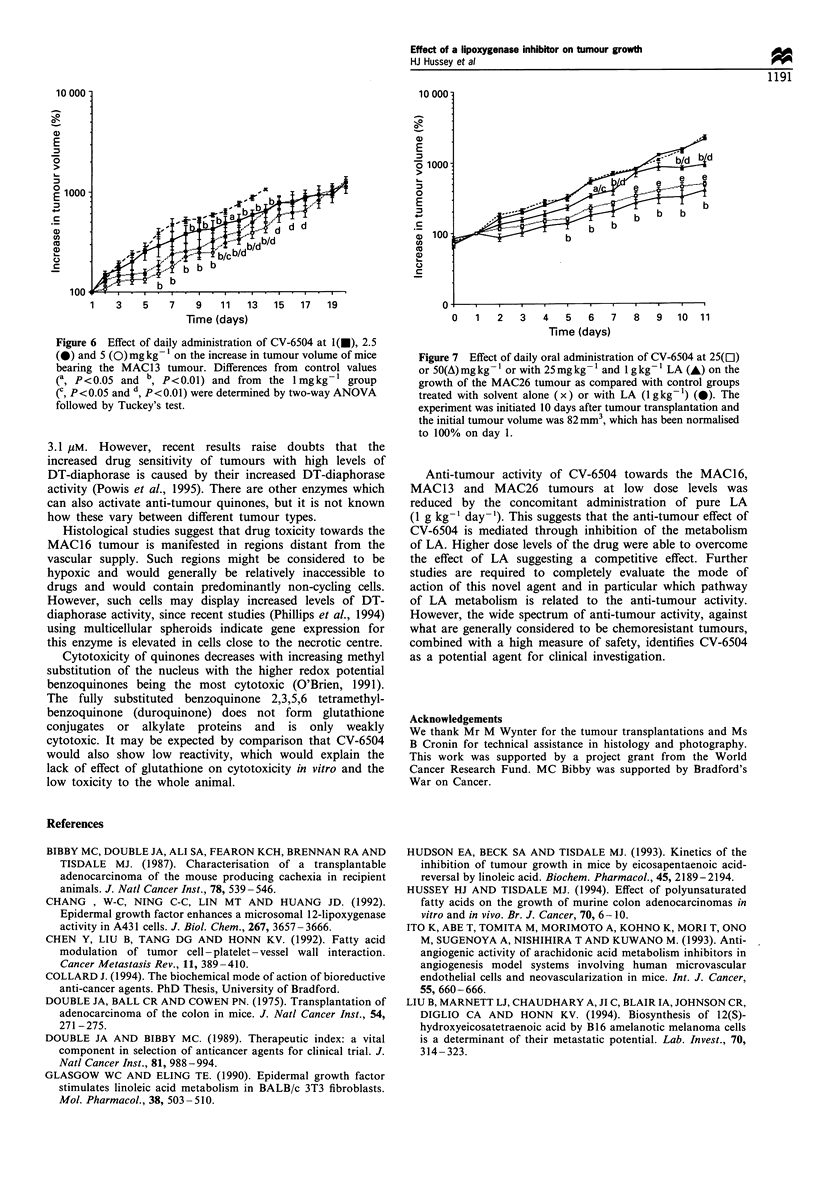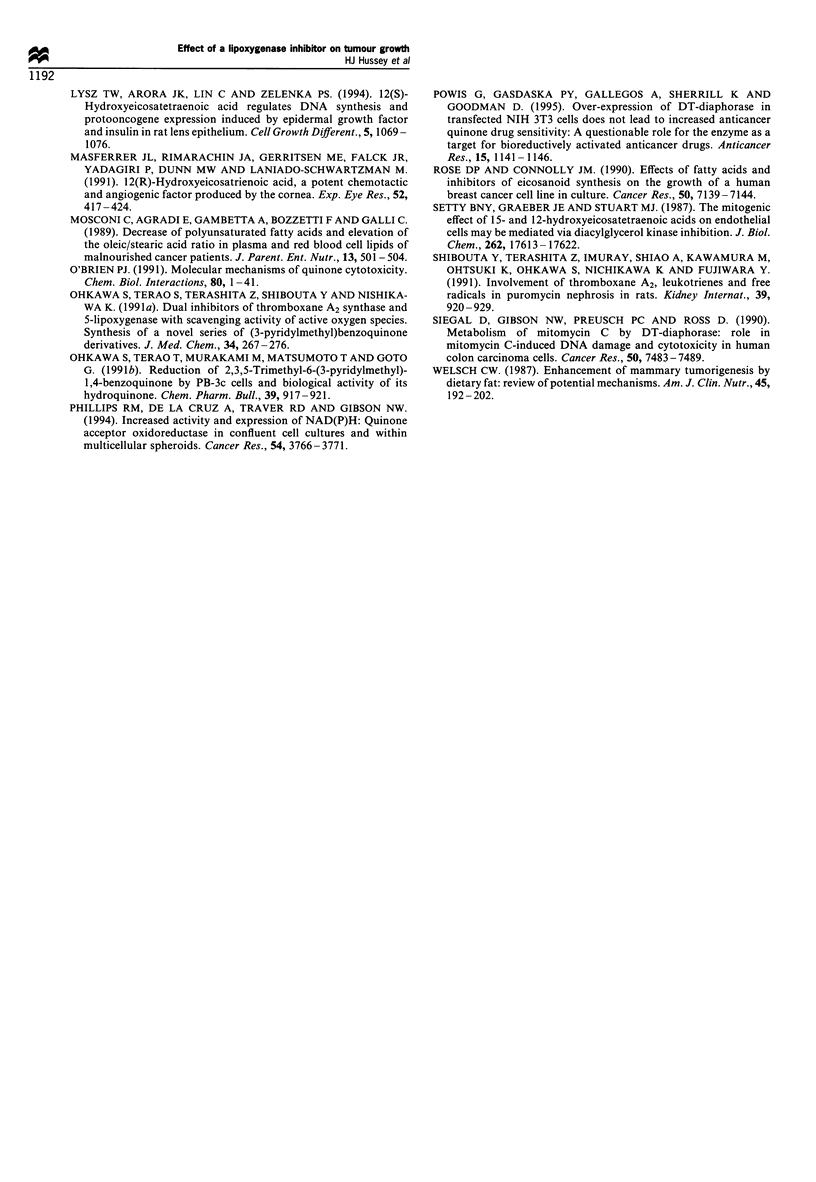# Novel anti-tumour activity of 2,3,5-trimethyl-6-(3-pyridylmethyl)-1,4- benzoquinone (CV-6504) against established murine adenocarcinomas (MAC).

**DOI:** 10.1038/bjc.1996.229

**Published:** 1996-05

**Authors:** H. J. Hussey, M. C. Bibby, M. J. Tisdale

**Affiliations:** Pharmaceutical Sciences Institute, Aston University, Birmingham, UK.

## Abstract

**Images:**


British Journal of Cancer (1996) 73, 1187-1192

© 1996 Stockton Press All rights reserved 0007-0920/96 $12.00

### Novel anti-tumour activity of 2,3,5-trimethyl-6-(3-pyridylmethyl)-1,4-benzoquinone (CV-6504) against established murine adenocarcinomas (MAC)

HJ Hussey[1], MC Bibby[2] and MJ Tisdale[1]

[1]*Pharmaceutical Sciences Institute, Aston University, Birmingham B4 7ET, UK;* [2]*Clinical Oncology Unit, University of Bradford, Bradford BD7 1DP, UK.*

**Summary** 2,3,5-Trimethyl-6-(3-pyridylmethyl)1,4-benzoquinone (CV-6504), an inhibitor of 5-lipoxygenase and thromboxane $A_2$ synthase and a scavenger of active oxygen species, has been shown to exhibit profound anti-tumour activity against three established murine adenocarcinomas (MACs) that are generally refractory to standard cytotoxic agents. For the cachexia-inducing MAC16 tumour, optimal anti-tumour activity was seen at dose levels of 10 and 25 mg $kg^{-1}$ $day^{-1}$, together with a reversal of cachexia and a doubling of the time to sacrifice of the animals through cachexia from 8 days to 17 days. The remaining tumour fragments showed extensive necrosis in regions distal from the blood supply. Growth of the MAC13 tumour was also effectively suppressed at dose levels between 5 and 50 mg $kg^{-1}$ $day^{-1}$, resulting in a specific growth delay between 1.0 and 1.2. Growth of the MAC26 tumour was also inhibited in a concentration-related manner, with doses of 25-50 mg $kg^{-1}$ $day^{-1}$ being optimal. Anti-tumour activity towards all three tumours at low dose levels of CV-6504 was effectively suppressed by concurrent administration of linoleic acid (1 g $kg^{-1}$ $day^{-1}$), suggesting that inhibition of linoleate metabolism was responsible for the anti-tumour effect. Tumour sensitivity may be correlated with increased DT-diaphorase levels that are required to metabolise CV-6504 to the active hydroquinone, which inhibits 5-lipoxygenase activity.

**Keywords:** anti-tumour; lipoxygenase inhibitor; quinone; murine adenocarcinoma

There is a growing body of evidence suggesting a role for polyunsaturated fatty acids (PUFAs), and in particular linoleic acid (LA) and arachidonic (AA) acid, as tumour promoters (Welsch, 1987) and as regulators of the growth of solid tumours (Hussey and Tisdale, 1994) and their metastasis (Chen *et al.*, 1992). The anti-proliferative but not the anti-cachectic effect of eicosapentaenoic acid (EPA) against the MAC16 tumour was reversed by concurrent administration of LA (Hudson *et al.*, 1993), suggesting that LA released from adipose tissue during the process of cachexia may be important for tumour growth. Significantly lower levels of LA as a percentage of total fatty acids were observed in plasma phospholipids, and cholesterol esters and in red blood cell phospholipids in patients with cachexia (Mosconi *et al.*, 1989) suggesting that human tumours may also be dependent on this essential fatty acid.

Studies *in vitro* (Hussey and Tisdale, 1994; Rose and Connolly, 1990) have suggested that metabolism of PUFA through the lipoxygenase pathway may be important for stimulation of tumour growth. Lipoxygenases constitute a family of closely related non-haeme iron-containing dioxygenases that convert PUFA to the corresponding hydroperoxy derivatives. In mammalian cells at least four types of lipoxygenase can be discriminated on the basis of the carbon atom of the substrate molecule (AA) at which oxygen is introduced: the 5-lipoxygenase, two 12-lipoxygenases (platelet type and leucocyte type) and the 15-lipoxygenases. Metabolism through the 12-lipoxygenase pathway may be most important for tumour growth and metastasis. Thus 12-(S)-hydroxyeicosatetraenoic acid (12(S)-HETE) has been suggested (Liu *et al.*, 1994) as a determinant of the metastatic potential of tumour cells and may be a crucial target for intervention in metastasis. 12(S)-HETE has been shown to stimulate DNA synthesis in fetal bovine aortic endothelial cells (Setty *et al.*, 1987) and to regulate expression of the proto-oncogenes c-*fos* and c-*myc* in rat lens epithelial cells (Lysz *et al.*, 1994), whereas 12(R)-HETE formed from AA by a cytochrome P450-dependent pathway caused neovascularisation of the cornea (Masferrer *et al.*, 1991). This could explain why inhibitors of the AA cascade exert anti-angiogenic activity in an *in vivo* model system of tumour angiogenesis in mice and inhibit tube formation *in vitro* (Ito *et al.*, 1993). Growth factors such as epidermal growth factor (EGF) enhance 12-HETE formation from AA (Chang *et al.*, 1992) and the hydroxy and hydroperoxy metabolites of LA enhance EGF-stimulated [$^3$H]thymidine incorporation in Balb/c 3T3 cells (Glasgow and Eling, 1990), suggesting that these metabolites may be part of the signal transduction cascade initiated by EGF.

These results suggest that inhibitors of the lipoxygenase pathways may provide useful new agents to inhibit tumour growth and metastasis. A number of inhibitors have been synthesised, mainly of the 5-lipoxygenase pathway. The present report concerns the novel anti-tumour activity of 2,3,5-trimethyl-6-(3-pyridylmethyl)-1,4-benzoquinone (CV-6504) against established colon adenocarcinomas of the MAC series which are refractory to standard cytotoxic agents (Double and Bibby, 1989) and which require PUFA for growth stimulation (Hussey and Tisdale, 1994). CV-6504 has inhibitory activity against both 5-lipoxygenase and thromboxane $A_2$ synthase as well as scavenging activity against active oxygen species (Ohkawa *et al.*, 1991*a,b*). The agent has undergone clinical investigation for the treatment of chronic glomerular nephritis.

### Materials and methods

### *Animals*

Pure-strain male NMRI mice were obtained from our own inbred colony under conventional conditions and were fed a rat and mouse breeding diet of which 50% of the energy was supplied by carbohydrate and 11.5% as fat. Linoleic acid comprised 4.5% of the fat giving a daily consumption of 45 mg (Hussey and Tisdale, 1994). The transplantable mouse

Correspondence: MJ Tisdale
Received 6 September 1995; revised 20 December 1995; accepted 21 December 1995

adenocarcinomas of the colon (MACs) were originally produced by prolonged administration of 1,2-dimethylhydrazine (Double *et al.*, 1975). Three tumours of this series were used in the present study, MAC16, MAC26 and MAC13 which were transplanted subcutaneously by trocar fragments into the flank. The MAC16 tumour is associated with cachexia which appears 9 to 12 days after transplantation (Bibby *et al.*, 1987). In all cases therapy was initiated 9–12 days after transplantation when the tumour became palpable and in the case of the MAC16 tumour weight loss had started to occur. Mice were randomised into groups of 8–10. CV-6504 was supplied by Takeda Chemical Industries, Osaka, Japan and was administered p.o. daily in aqueous solution (0.1 ml). Control mice received water alone (0.1 ml). Tumour dimensions were measured daily by means of calipers and the volume was calculated from the formula:

$$\frac{\text{length} \times (\text{width})^2}{2}$$

Animals were killed if the tumour ulcerated, weight loss reached 25 to 30% of the original body weight, the tumour weight reached 10% of the host weight or the animals became moribund as agreed by the Co-ordinating Committee on Cancer Research of the UK for the welfare of animals with neoplasms. The experiment was repeated three times with the MAC16 tumour and twice with the MAC13 and MAC26 tumours. The results shown in Figures 1, 4, 6 and 7 are representative of a single experiment.

### *Cell lines*

MAC13, MAC26 and MAC16 cell lines were derived from the solid tumours and maintained *in vitro* in RPMI-1640 medium supplemented with either 10% (MAC13 and MAC26) or 5% (MAC16) fetal calf serum at 37°C under an atmosphere of 5% carbon dioxide in air. For cell growth assays cells were seeded either at 0.5 (MAC13 and MAC26) or $2.0 \times 10^4$ cells per well (MAC16) and left for 2 h before drug addition. Cell counts were made 72 h after drug addition using a ZM Coulter counter.

### *Histology*

Tumour tissue from both control and treated animals was processed for histological examination. Tumours were fixed in Bouin's fluid, dehydrated in ethanol and embedded in paraffin wax. Sections (5 $\mu$m) were stained with haematoxylin and eosin.

### *Statistical analysis*

The data were statistically evaluated using two-way analysis of variance.

### Results

CV-6504 was a potent inhibitor of the growth of the MAC cell lines *in vitro*, the $IC_{50}$ values (concentration in $\mu$M reducing cell numbers by 50% 72 h after seeding) being for MAC16, $3 \pm 1$, for MAC26 $7 \pm 1$ and for MAC13 $3 \pm 1$ $\mu$M. These values were about 10-fold lower than previously observed with the cyclooxygenase inhibitor indomethacin (Hussey and Tisdale, 1994). The $IC_{50}$ value to MAC16 was not increased by extracellular glutathione levels between 1 and 100 $\mu$M, suggesting that it did not react with glutathione *in vitro*.

The MAC tumours are relatively slow growing *in vivo* with volume doubling times of 4 days (MAC16), 3.5 days (MAC13) and 3 days (MAC26) and show varying degrees of differentiation. The effect of CV-6504 on the growth of the MAC16 tumour and the effect on host body weight loss is shown in Figure 1. Therapy was initiated 9 days after transplantation when the tumour volume was 100 mm³. The volume has been normalised to 100% and the starting point of the experiment is shown as day 1 in Figure 1a. Both tumour growth and host weight loss were inhibited in a dose-related manner with no significant difference being observed with doses of 10 and 25 mg kg$^{-1}$ day$^{-1}$. Both doses lead to an increased time to sacrifice of the animals through cachexia without any evidence of toxicity. In fact as shown in Figure 1b, since cachexia was totally suppressed the animals actually increased in body weight. The effect on tumour volume was more pronounced at larger tumour volumes for the two higher dose levels of CV-6504. Thus for CV-6504 at 25 mg kg$^{-1}$ the specific growth delay increased from 0.75 after one doubling to 2.2 after two doublings. Likewise at 10 mg kg$^{-1}$ the specific growth delay was only 0.75 after one doubling, but the tumour never reached two doublings during the 17 days of the experiment. Tumour growth inhibition, reversal of cachexia and prolongation of survival produced by CV-6504 at 10 mg kg$^{-1}$ were reduced when LA (1 g kg$^{-1}$) was administered concomitantly on a daily basis. Histopathological examination of tumour fragments remaining after 17 days administration of CV-6504 (10 mg kg$^{-1}$ day$^{-1}$) (Figure 2) showed evidence of massive necrosis with few viable tumour cells, fibrosis and lymphocytic infiltration. Tumour vasculature remained intact and there was evidence of viable tumour cells around the blood vessels (Figure 3a). There was extensive necrosis and a fibrous capsule which walled in the few remaining viable cells and areas of necrosis (Figure 3b). Control MAC16 tumours showed little necrosis and no capsule (Figure 3c).

**Figure 1** Effect of oral administration of CV-6504 on tumour growth (**a**) and host body weight (**b**) in male mice transplanted with the MAC16 tumour. The experiment was initiated 9 days after tumour transplantation (day 1) and the absolute tumour volume was 100 mm³. Both tumour volume and host body weight were normalised to 100% on day 1. CV-6504 was administered p.o. on a daily basis at doses of 5 (○), 10 (▲) and 25 (□) mg kg$^{-1}$ in water, whereas control animals (×) received water alone. Differences from control values [a], $P<0.05$ and [b], $P<0.01$ were determined using two-way ANOVA followed by Tuckey's test.

**Figure 2** Sections through the MAC16 tumour from control animals at day 6 (**a**) or after 17 days on CV-6504 at 10 mg kg$^{-1}$ day$^{-1}$ (**b**).

**Figure 3** (**a**) Section through the MAC16 tumour after 17 days treatment with CV-6504 at 10 mg kg$^{-1}$ day$^{-1}$ showing viable tumour cells around the blood vessels and the presence of a fibrous capsule (**b**). Section through a control MAC16 tumour (**c**) showing the absence of the fibrous capsule.

Growth of the MAC13 tumour was also effectively suppressed by administration of CV-6504 at dose levels between 5 and 50 mg kg$^{-1}$ day$^{-1}$ (Figure 4a). Lower dose levels (5 and 10 mg kg$^{-1}$ day$^{-1}$) appeared to produce more effective tumour suppression and a higher specific growth delay than higher doses (Table I). Growth inhibition produced by CV-6504 at 5 mg kg$^{-1}$ day$^{-1}$ was reduced by co-administration of LA (1 g kg$^{-1}$ day$^{-1}$) (Figure 4b) but this effect was counteracted by increasing the dose of CV-6504 to 10 mg kg$^{-1}$ day$^{-1}$ (Figure 4c). Histological examination of sections of treated tumour compared with control revealed significant morphological effects. Control sections (Figure 5a) demonstrated the typical glandular pattern of this tumour, whereas treatment resulted in central necrosis (Figure 5b, c) with evidence of lymphocyte infiltration. The effect of lower dose levels of CV-6504 on growth of the MAC13 tumour was addressed in a second experiment (Figure 6). This showed that a dose level of 5 mg kg$^{-1}$ day$^{-1}$ produced a significantly greater growth inhibition of the MAC13 tumour than did the lower dose levels.

Growth of the MAC26 tumour was also inhibited by CV-6504 in a dose-related manner (Figure 7). This tumour was less sensitive to CV-6504 than the other MAC tumours and dose levels of 25 and 50 mg kg$^{-1}$ day$^{-1}$ were found to be optimal (Table I). Again a reduction of the anti-tumour effect of CV-6504 (25 mg kg$^{-1}$ day$^{-1}$) was observed with concurrent administration of LA (1 g kg$^{-1}$ day$^{-1}$).

### Discussion

Previous studies (Double and Bibby, 1989) have demonstrated the MAC tumour series to be generally refractory to standard cytotoxic agents, with the MAC13 probably being the most responsive. In contrast with this general lack of responsiveness the present study demonstrates profound anti-tumour activity of CV-6504 against three established MAC tumours in an *in vivo* assay. For most anti-tumour agents activity is usually only demonstrated at the maximum tolerated dose. Thus the therapeutic index for the standard nitrosoureas against chemoresponsive tumours such as the MAC13 is $<1$ and even against the L1210 leukaemia is no higher than 7 (Double and Bibby, 1989). This study demonstrates optimal anti-tumour activity of CV-6504 against the MAC16 tumour at 10 mg kg$^{-1}$ day$^{-1}$, against the MAC13 tumours at 5–10 mg kg$^{-1}$ day$^{-1}$ and against the MAC26 tumour at 50 mg kg$^{-1}$ day$^{-1}$. Chronic toxicity studies showed that the non-toxic dosage level in male mice was 100 mg kg$^{-1}$ day$^{-1}$. Thus CV-6504 not only demonstrated profound anti-tumour activity against previously chemoresistant animal models, but did so at a dose with a high margin of safety. This dose is comparable with that previously employed for the inhibition of puromycin

**Figure 4** Effect of oral administration of CV-6504 on tumour growth of mice transplanted with the MAC13 tumour in the absence (**a**) or presence (**b** and **c**) of LA ($1\,g\,kg^{-1}\,day^{-1}$). The experiment was initiated 9 days after tumour transplantation and the initial tumour volume was $91 \pm 10\,mm^3$, which has been normalised to 100% on day 1. Animals were treated daily with CV-6504 at 5 (○), 10 (▲), 25 (□) or 50 (Δ) $mg\,kg^{-1}$ in water while control animals (×) received water alone; or (**b** and **c**) LA (●) plus (**b**) CV-6504 at 5 and (**c**) $10\,mg\,kg^{-1}$ (■). Differences ([a], $P=0.05$ and [b], $P=0.01$) from control tumour volume given water alone and ([c], $P=0.05$ and [d], $P=0.01$) given LA were determined by two-way ANOVA followed by Tuckey's test.

**Table I** Specific growth delay induced by CV-6504[a]

| *CV-6504 (mg/kg$^{-1}$)* | *MAC16* | *Tumour type MAC13* | *MAC26* |
|---|---|---|---|
| 5 | 0.7 | 1.2 | ND |
| 10 | –[b] | 1.2 | ND |
| 25 | 2.2 | 1.0 | 0.8 |
| 50 | ND | 1.0 | 1.2 |

[a]Determined after two tumour doublings.[b]At this dose level the tumour did not reach two doublings. ND, not determined.

aminonucleoside nephrosis in rats (Shibouta *et al.*, 1991).

The MAC16 tumour was found to be more responsive to CV-6504 than the MAC26 tumour (Table I). Mitomycin C and doxorubicin are the only two other chemotherapeutic agents which are more effective against MAC16 than

**Figure 5** Histological appearance of control MAC13 tumour (**a**) and changes seen following treatment with oral CV-6504 at $25\,mg\,kg^{-1}\,day^{-1}$. (**b**) illustrates a large area of necrosis which was typical of treated tumours. The appearance of the necrotic changes is shown in more detail in (**c**).

MAC26 (Double and Bibby, 1989). These are also quinone-containing drugs that can undergo metabolic reduction and for mitomycin C there is evidence to suggest activation by DT-diaphorase (Siegal *et al.*, 1990). Previous studies on the mode of action of CV-6504 suggest a reduction to the hydroquinone by two electron-donating enzymes such as DT-diaphorase without the intermediary of a semiquinone radical (Ohkawa *et al.*, 1991*b*). Lipid peroxidation as well as 5-lipoxygenase activity is prevented by reducing the ferric iron in the active site of the enzyme to the ferrous (resting state) and unlike a number of anti-tumour quinones, CV-6504 is capable of scavenging active oxygen species. The increased sensitivity of the MAC16 to CV-6504 could be correlated with an increased DT-diaphorase activity that has been reported to be 16-fold higher than in the MAC26 tumour (Collard, 1994) with the MAC13 tumour displaying a similar low value. Certainly CV-6504 is an excellent substrate for both human and mouse DT-diaphorase with a $k_{cat}$ of $6 \times 10^4\,min^{-1}$ and $K_m$ of $50\,\mu M$ (RJ Knox, Personal communication). These results can be compared with the classic DT-diaphorase substrate menadione, which has the same $k_{cat}$, but a $K_m$ of only

**Figure 6** Effect of daily administration of CV-6504 at 1(■), 2.5 (●) and 5 (○) mg kg$^{-1}$ on the increase in tumour volume of mice bearing the MAC13 tumour. Differences from control values ($^a$, $P<0.05$ and $^b$, $P<0.01$) and from the 1 mg kg$^{-1}$ group ($^c$, $P<0.05$ and $^d$, $P<0.01$) were determined by two-way ANOVA followed by Tuckey's test.

**Figure 7** Effect of daily oral administration of CV-6504 at 25(□) or 50(Δ) mg kg$^{-1}$ or with 25 mg kg$^{-1}$ and 1 g kg$^{-1}$ LA (▲) on the growth of the MAC26 tumour as compared with control groups treated with solvent alone (×) or with LA (1 g kg$^{-1}$) (●). The experiment was initiated 10 days after tumour transplantation and the initial tumour volume was 82 mm$^3$, which has been normalised to 100% on day 1.

3.1 μM. However, recent results raise doubts that the increased drug sensitivity of tumours with high levels of DT-diaphorase is caused by their increased DT-diaphorase activity (Powis *et al.*, 1995). There are other enzymes which can also activate anti-tumour quinones, but it is not known how these vary between different tumour types.

Histological studies suggest that drug toxicity towards the MAC16 tumour is manifested in regions distant from the vascular supply. Such regions might be considered to be hypoxic and would generally be relatively inaccessible to drugs and would contain predominantly non-cycling cells. However, such cells may display increased levels of DT-diaphorase activity, since recent studies (Phillips *et al.*, 1994) using multicellular spheroids indicate gene expression for this enzyme is elevated in cells close to the necrotic centre.

Cytotoxicity of quinones decreases with increasing methyl substitution of the nucleus with the higher redox potential benzoquinones being the most cytotoxic (O'Brien, 1991). The fully substituted benzoquinone 2,3,5,6 tetramethylbenzoquinone (duroquinone) does not form glutathione conjugates or alkylate proteins and is only weakly cytotoxic. It may be expected by comparison that CV-6504 would also show low reactivity, which would explain the lack of effect of glutathione on cytotoxicity *in vitro* and the low toxicity to the whole animal.

Anti-tumour activity of CV-6504 towards the MAC16, MAC13 and MAC26 tumours at low dose levels was reduced by the concomitant administration of pure LA (1 g kg$^{-1}$ day$^{-1}$). This suggests that the anti-tumour effect of CV-6504 is mediated through inhibition of the metabolism of LA. Higher dose levels of the drug were able to overcome the effect of LA suggesting a competitive effect. Further studies are required to completely evaluate the mode of action of this novel agent and in particular which pathway of LA metabolism is related to the anti-tumour activity. However, the wide spectrum of anti-tumour activity, against what are generally considered to be chemoresistant tumours, combined with a high measure of safety, identifies CV-6504 as a potential agent for clinical investigation.

**Acknowledgements**

We thank Mr M Wynter for the tumour transplantations and Ms B Cronin for technical assistance in histology and photography. This work was supported by a project grant from the World Cancer Research Fund. MC Bibby was supported by Bradford's War on Cancer.

### References

BIBBY MC, DOUBLE JA, ALI SA, FEARON KCH, BRENNAN RA AND TISDALE MJ. (1987). Characterisation of a transplantable adenocarcinoma of the mouse producing cachexia in recipient animals. *J. Natl Cancer Inst.*, **78**, 539–546.

CHANG , W-C, NING C-C, LIN MT AND HUANG JD. (1992). Epidermal growth factor enhances a microsomal 12-lipoxygenase activity in A431 cells. *J. Biol. Chem.*, **267**, 3657–3666.

CHEN Y, LIU B, TANG DG AND HONN KV. (1992). Fatty acid modulation of tumor cell–platelet–vessel wall interaction. *Cancer Metastasis Rev.*, **11**, 389–410.

COLLARD J. (1994). The biochemical mode of action of bioreductive anti-cancer agents. PhD Thesis, University of Bradford.

DOUBLE JA, BALL CR AND COWEN PN. (1975). Transplantation of adenocarcinoma of the colon in mice. *J. Natl Cancer Inst.*, **54**, 271–275.

DOUBLE JA AND BIBBY MC. (1989). Therapeutic index: a vital component in selection of anticancer agents for clinical trial. *J. Natl Cancer Inst.*, **81**, 988–994.

GLASGOW WC AND ELING TE. (1990). Epidermal growth factor stimulates linoleic acid metabolism in BALB/c 3T3 fibroblasts. *Mol. Pharmacol.*, **38**, 503–510.

HUDSON EA, BECK SA AND TISDALE MJ. (1993). Kinetics of the inhibition of tumour growth in mice by eicosapentaenoic acid-reversal by linoleic acid. *Biochem. Pharmacol.*, **45**, 2189–2194.

HUSSEY HJ AND TISDALE MJ. (1994). Effect of polyunsaturated fatty acids on the growth of murine colon adenocarcinomas *in vitro* and *in vivo*. *Br. J. Cancer*, **70**, 6–10.

ITO K, ABE T, TOMITA M, MORIMOTO A, KOHNO K, MORI T, ONO M, SUGENOYA A, NISHIHIRA T AND KUWANO M. (1993). Anti-angiogenic activity of arachidonic acid metabolism inhibitors in angiogenesis model systems involving human microvascular endothelial cells and neovascularization in mice. *Int. J. Cancer*, **55**, 660–666.

LIU B, MARNETT LJ, CHAUDHARY A, JI C, BLAIR IA, JOHNSON CR, DIGLIO CA AND HONN KV. (1994). Biosynthesis of 12(S)-hydroxyeicosatetraenoic acid by B16 amelanotic melanoma cells is a determinant of their metastatic potential. *Lab. Invest.*, **70**, 314–323.

LYSZ TW, ARORA JK, LIN C AND ZELENKA PS. (1994). 12(S)-Hydroxyeicosatetraenoic acid regulates DNA synthesis and protooncogene expression induced by epidermal growth factor and insulin in rat lens epithelium. *Cell Growth Different.*, **5**, 1069–1076.

MASFERRER JL, RIMARACHIN JA, GERRITSEN ME, FALCK JR, YADAGIRI P, DUNN MW AND LANIADO-SCHWARTZMAN M. (1991). 12(R)-Hydroxyeicosatrienoic acid, a potent chemotactic and angiogenic factor produced by the cornea. *Exp. Eye Res.*, **52**, 417–424.

MOSCONI C, AGRADI E, GAMBETTA A, BOZZETTI F AND GALLI C. (1989). Decrease of polyunsaturated fatty acids and elevation of the oleic/stearic acid ratio in plasma and red blood cell lipids of malnourished cancer patients. *J. Parent. Ent. Nutr.*, **13**, 501–504.

O'BRIEN PJ. (1991). Molecular mechanisms of quinone cytotoxicity. *Chem. Biol. Interactions*, **80**, 1–41.

OHKAWA S, TERAO S, TERASHITA Z, SHIBOUTA Y AND NISHIKAWA K. (1991*a*). Dual inhibitors of thromboxane $A_2$ synthase and 5-lipoxygenase with scavenging activity of active oxygen species. Synthesis of a novel series of (3-pyridylmethyl)benzoquinone derivatives. *J. Med. Chem.*, **34**, 267–276.

OHKAWA S, TERAO T, MURAKAMI M, MATSUMOTO T AND GOTO G. (1991*b*). Reduction of 2,3,5-Trimethyl-6-(3-pyridylmethyl)-1,4-benzoquinone by PB-3c cells and biological activity of its hydroquinone. *Chem. Pharm. Bull.*, **39**, 917–921.

PHILLIPS RM, DE LA CRUZ A, TRAVER RD AND GIBSON NW. (1994). Increased activity and expression of NAD(P)H: Quinone acceptor oxidoreductase in confluent cell cultures and within multicellular spheroids. *Cancer Res.*, **54**, 3766–3771.

POWIS G, GASDASKA PY, GALLEGOS A, SHERRILL K AND GOODMAN D. (1995). Over-expression of DT-diaphorase in transfected NIH 3T3 cells does not lead to increased anticancer quinone drug sensitivity: A questionable role for the enzyme as a target for bioreductively activated anticancer drugs. *Anticancer Res.*, **15**, 1141–1146.

ROSE DP AND CONNOLLY JM. (1990). Effects of fatty acids and inhibitors of eicosanoid synthesis on the growth of a human breast cancer cell line in culture. *Cancer Res.*, **50**, 7139–7144.

SETTY BNY, GRAEBER JE AND STUART MJ. (1987). The mitogenic effect of 15- and 12-hydroxyeicosatetraenoic acids on endothelial cells may be mediated via diacylglycerol kinase inhibition. *J. Biol. Chem.*, **262**, 17613–17622.

SHIBOUTA Y, TERASHITA Z, IMURAY, SHIAO A, KAWAMURA M, OHTSUKI K, OHKAWA S, NICHIKAWA K AND FUJIWARA Y. (1991). Involvement of thromboxane $A_2$, leukotrienes and free radicals in puromycin nephrosis in rats. *Kidney Internat.*, **39**, 920–929.

SIEGAL D, GIBSON NW, PREUSCH PC AND ROSS D. (1990). Metabolism of mitomycin C by DT-diaphorase: role in mitomycin C-induced DNA damage and cytotoxicity in human colon carcinoma cells. *Cancer Res.*, **50**, 7483–7489.

WELSCH CW. (1987). Enhancement of mammary tumorigenesis by dietary fat: review of potential mechanisms. *Am. J. Clin. Nutr.*, **45**, 192–202.